# The Forward Testing Effect is Reliable and Independent of Learners’ Working Memory Capacity

**DOI:** 10.5334/joc.82

**Published:** 2019-08-29

**Authors:** Bernhard Pastötter, Christian Frings

**Affiliations:** 1 Department of Psychology, University of Trier, DE

**Keywords:** Memory, Long-term memory, Working memory, Learning

## Abstract

The forward testing effect refers to the finding that retrieval practice of previously studied information enhances learning and retention of subsequently studied other information. While most of the previous research on the forward testing effect examined group differences, the present study took an individual differences approach to investigate this effect. Experiment 1 examined whether the forward effect has test-retest reliability between two experimental sessions. Experiment 2 investigated whether the effect is related to participants’ working memory capacity. In both experiments (and each session of Experiment 1), participants studied three lists of items in anticipation of a final cumulative recall test. In the testing condition, participants were tested immediately on lists 1 and 2, whereas in the restudy condition, they restudied lists 1 and 2. In both conditions, participants were tested immediately on list 3. On the group level, the results of both experiments demonstrated a forward testing effect, with interim testing of lists 1 and 2 enhancing immediate recall of list 3. On the individual level, the results of Experiment 1 showed that the forward effect on list 3 recall has moderate test-retest reliability between two experimental sessions. In addition, the results of Experiment 2 showed that the forward effect on list 3 recall does not depend on participants’ working memory capacity. These findings suggest that the forward testing effect is reliable at the individual level and affects learners at a wide range of working memory capacities alike. The theoretical and practical implications of the findings are discussed.

## Introduction

Tests are commonly used to evaluate learning. However, tests can also be used to enhance learning and retention. This has been demonstrated in both basic research in the laboratory (for reviews, see Karpicke, 2017; Roediger & Butler, 2011; Rowland, 2014) and applied research in educational contexts (for reviews, see Adesope, Trevisan, & Sundararajan, 2017; Dunlosky, Rawson, Marsh, Nathan, & Willingham, 2013; Moreira, Pinto & Starling, 2019). Thereby, testing can have both direct and indirect effects on long-term learning and retention (Roediger & Karpicke, 2006a). A prominent *direct* benefit of testing is the (backward) testing effect, which refers to the finding that when participants (repeatedly) retrieve versus restudy previously studied item material, the retrieved items are better recalled on a delayed test than the restudied items (e.g., Hogan & Kintsch, 1971; Roediger & Karpicke, 2006b). The backward testing effect is a robust phenomenon that generalizes across a wide range of materials and experimental settings (see Karpicke, 2017). Different mechanisms have been suggested to contribute to the backward testing effect. For instance, retrieval practice, more than restudy, may promote semantic elaboration (Carpenter, 2009) and/or may enhance contextual processing of the information (Karpicke, Lehman, & Aue, 2014), thus enhancing learning and memory accessibility of the practiced information.

In addition, there are *indirect* benefits of testing. Actually, it is these indirect benefits of testing that students typically aim for when practicing retrieval on their own, not the direct benefits of testing (Karpicke, Butler, & Roediger, 2009). For instance, retrieval practice of previously studied material potentiates relearning of the practiced information, a finding that has been termed test-potentiated learning (Arnold & McDermott, 2013; Izawa, 1971; see also Tempel & Frings, 2018). The typical experiment consists of a study phase, a restudy phase, and a final test phase. Critically, between study and restudy phases, it is manipulated whether the material is tested or not. The typical finding is that interpolated testing promotes relearning of the studied material and enhances memory performance in the final test. On the other hand, retrieval practice also potentiates subsequent learning of other information, a finding that has been referred to as forward testing effect (Pastötter & Bäuml, 2014) or test-potentiated new learning (Chan, Meissner, & Davis, 2018). The forward testing effect is particularly striking because it is on the learning of *new* information that is not necessarily related to the previously studied (and tested) item material (see Yang, Chew, Sun, & Shanks, 2019). The forward testing effect can be studied in interim-testing tasks: Participants study several (e.g., three) lists of items in anticipation of a final cumulative recall test. Between the study of lists, participants are either tested on the most recent list (i.e., list 1 or list 2) in a recall test, or restudy the items of the most recent list. After study of all lists, participants are tested on the last list (i.e., list 3), which is the critical list. The typical finding is that interim testing of lists 1 and 2 enhances recall of list 3 and reduces the number of prior-list intrusions in the list 3 recall test (e.g., Bäuml & Kliegl, 2013; Szpunar, McDermott, & Roediger, 2008; Weinstein, McDermott, & Szpunar, 2011; Yang et al., 2019). The forward testing effect has been observed with different item materials (e.g., words, pictures, texts, videos), for different numbers of to-be-studied item lists (e.g., two lists, three lists, five lists) and for different types of recall tasks used in the interim tests (e.g., cued recall, free recall; for reviews, see Pastötter & Bäuml, 2014; Yang, Potts, & Shanks, 2018; for a meta-analysis on the forward testing effect, see Chan et al., 2018).

Both encoding and retrieval accounts have been put forth to account for the forward testing effect (see Yang et al., 2018, for a recent review). Retrieval accounts assume that recall testing between the study of lists promotes contextual list segregation, which may enhance list differentiation and reduce interference between lists at test (Bäuml & Kliegl, 2013; Szpunar et al., 2008). In contrast, encoding accounts assume that testing of previously studied lists improves encoding of subsequently studied item material. Specifically, it has been suggested that interim testing may reduce memory load (i.e., interference during encoding) and enhance attentional encoding, which makes the encoding of later lists as effective as the encoding of earlier lists (Pastötter et al., 2018; Pastötter, Schicker, Niedernhuber, & Bäuml, 2011), or a change in participants’ encoding strategy, which enhances elaborative encoding for the later lists compared to the earlier lists (Chan, Manley, Davis, & Szpunar, 2018; Wissman, Rawson, & Pyc, 2011; see also Garcia-Marques, Nunes, Marques, Carneiro, & Weinstein, 2015). In addition, motivational factors have been suggested that may mediate the encoding and/or retrieval benefits of testing. Accordingly, retrieval failures in tests may induce dissatisfaction in learners and thus motivate more effort toward subsequent encoding and/or retrieval of new information (Cho, Neely, Crocco, & Vitrano, 2017). Furthermore, interim testing may induce in learners a greater expectancy of additional tests, which may motivate more effort toward attentional encoding of new information (Weinstein, Gilmore, Szpunar, & McDermott, 2014). Thus, multiple encoding and retrieval factors and different mediators may contribute to the forward testing effect. Notably, the same factors may also contribute to test-potentiated learning, as has been suggested in a recent study by Cho et al. (2017). These authors demonstrated comparable indirect effects of interpolated testing on the relearning and final recall of previously studied, old items (test-potentiated learning) and the learning and recall of previously not studied, new items (forward testing effect).

Regarding individual-differences research on testing effects, a considerable amount of studies have examined possible influences of learners’ age and working memory capacity (WMC) on testing effects. This research has established both direct and indirect testing effects in children, young people, and older adults. For instance, children in (late) primary school benefit from the backward testing effect (Karpicke, Blunt, & Smith, 2016), the forward testing effect (Aslan & Bäuml, 2016), and test-potentiated learning (Lipowski, Pyc, Dunlosky, & Rawson, 2014), and even preschool children may benefit from some forms of retrieval practice (Kliegl, Abel, & Bäuml, 2018; see Fazio & Marsh, in press, for a review). In addition, several studies have established both backward (Meyer & Logan, 2013) and forward testing effects (Pastötter & Bäuml, 2019) and test-potentiated learning (Coane, 2013) in older adults, with the size of the effects comparable to younger adults. Furthermore, reliable testing effects were observed in memory-impaired patient groups, including patients with multiple sclerosis, traumatic brain injury, and Alzheimer’s disease (Balota, Duchek, Sergent-Marshall, & Roediger, 2006; Pastötter, Weber, & Bäuml, 2013; Sumowski, Chiaravalloti, & DeLuca, 2010; Sumowski, Wood et al., 2010; but see Pastötter, Eberle, Aue, & Bäuml, 2017).

Regarding the relationship between WMC and the benefits of retrieval practice, several studies have examined whether complex working memory tasks, such as the operation or symmetry span tasks, relate to the backward testing effect or test-potentiated learning. WMC is an interesting individual-differences variable in cognitive psychology because it accounts for a substantial portion of variance in a person’s general fluid intelligence and executive function (see Conway, Kane, & Engle, 2003; Foster et al., 2015; McCabe, Roediger, McDaniel, Balota, & Hambrick, 2010). The majority of these studies found no (direct) relationship between WMC and the backward testing effect effect (Agarwal, Finley, Rose, & Roediger, 2017; Aslan & Bäuml, 2011) or test-potentiated learning (Bertilsson, Wiklund-Hörnqvist, Stenlund, & Jonsson, 2017; Brewer & Unsworth, 2012; Minear, Coane, Boland, Cooney, & Albat, 2018; Tsu & Pu, 2012; Wiklund-Hörnqvist, Jonsson, & Nyberg, 2014; but see Agarwal et al., 2017). Notably, the study by Tse and Pu (2012) showed that a significant proportion of variance in test-potentiated learning could be predicted by an interaction between participants’ WMC and test anxiety scores. Thus, more indirect relationships between WMC and the benefits of retrieval practice may exist. To the best of our knowledge, no study has yet examined the relationship between the forward testing effect and participants’ WMC.

## Overview

In two experiments we investigated individual differences in the forward testing effect. In both experiments, participants went through both a testing and a restudy condition. In each condition, participants studied three lists of words, which they were asked to remember for a final recall test. In the testing condition, they were tested immediately on lists 1 and 2 after studying each single list, whereas in the restudy condition, they restudied lists 1 and 2 after initial study. In both conditions, participants were tested immediately on list 3 and finally on lists 1 and 2. The critical variables were correct recall and the number of prior-list intrusions in the immediate list 3 recall test. Individual forward-testing-effect recall and intrusion scores (shortly referred to as FTE recall and intrusion scores in the following) were determined by calculating individual differences between conditions. Experiment 1 examined the test-retest reliability of the forward testing effect, i.e., the correlation of FTE recall and intrusion scores between two experimental sessions. Experiment 2 examined the relationship between the forward testing effect and participants’ WMC, measured with complex WM span tasks. Because the reliability of a measure limits the correlation that can be observed with another measure (Nunally, 1970; Spearman, 1904), significant test-retest reliability of FTE scores in Experiment 1 was considered a necessary precondition for analyzing the relationship of these scores to participants’ WMC in Experiment 2. Based on the finding that test-potentiated learning is not related to learners’ WMC (Brewer & Unsworth, 2012; Wiklund-Hörnqvist et al., 2014) and the view that the same factors contribute to test-potentiated learning and the forward effect of testing (Cho et al., 2017), we hypothesized to find no significant correlation between the forward testing effect and participants’ WMC. To foreshadow the results, the data of Experiment 1 suggested sufficient reliability of the forward testing effect and thereby enabled us to analyze the potential relationship of the effect to individual WMC. In accordance with our hypothesis we did not find evidence for a correlation. Note that as we actually predicted a null correlation, Bayesian statistics are reported in addition to frequentist statistics.

## Experiment 1

The goal of Experiment 1 was to examine test-retest reliability of the forward testing effect. Participants took part in two sessions, which both consisted of a testing and a restudy condition. The two sessions were one week apart. Different items were used in the two sessions. FTE recall and intrusion scores were calculated for both sessions and test-retest reliabilities were estimated using Intraclass Correlation Coefficients (ICC; see Hedge, Powell, & Sumner, 2018; Parsons, Kruijt, & Fox, 2018).

### Method

#### Participants

Required sample size for the test-retest reliability analysis in Experiment 1 was calculated based on methods suggested by Walter, Eliasziw, and Donner (1998). Given *α* = 0.05 and a desired power of 1 – *β* = 0.95 to observe a fair level of reliability with ICC of 0.3 (or larger), a required sample size of 57 subjects was calculated. Note that ICCs of 0.8, 0.6, and 0.4 are typically interpreted as excellent, good/substantial, and moderate levels of reliability, respectively (Fleiss, 1981; Landis & Koch, 1977).

Sixty-four students from the University of Trier participated in Experiment 1 (mean age: 22.4 years, SD = 2.5 years; 24 females, 40 males). All participants successfully completed both sessions. The study was carried out in accordance with the recommendations of the Declaration of Helsinki and approved by the local ethical review committee at the University of Trier (reference number: 50/2017). All participants gave written informed consent.

#### Material

The material was taken from a study by Pastötter, Kliegl, and Bäuml (2012; Exp. 2). It consisted of 144 unrelated German nouns of medium frequency and word length of 4 to 8 letters, which were drawn from CELEX database (Duyck, Desmet, Verbeke, & Brysbaert, 2004). The 144 items were randomly assigned to twelve 12-item lists. Six lists were used in session 1, the other six lists in session 2. In each session, three lists were assigned to the testing condition, the other three lists were assigned to the restudy condition. Thus, different items were used in the two sessions.

#### Procedure

**Figure 1:**
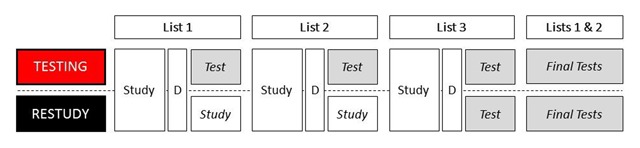
Procedure of the episodic memory task. In both the testing and the restudy condition, participants studied three lists of items. Each list consisted of 12 words and was followed by a short distractor (D). List 3 was tested immediately (after the distractor) in both conditions. Lists 1 and 2 were also tested immediately in the testing condition, but were restudied in the restudy condition. After immediate recall of list 3, lists 1 and 2 were tested in final recall tests.

The two sessions were one week apart. Participants were retested in session 2 at about the same time as they were tested in session 1, with maximum deviation of two hours between sessions. In both sessions, participants took part in both the testing and the restudy condition, with order of conditions counterbalanced across participants. In both conditions, participants studied three 12-item lists (see Figure 1). Items of each list were visually presented in random order in the middle of a computer screen with a presentation rate of 3.75 sec (3 sec item presentation, 0.75 sec blank screen). Subsequent to the presentation of each list (45 sec), all participants solved simple mathematics (e.g., 4 * 8) for 30 sec as a distractor. Experimental conditions differed in inter-list activity that followed the distractors after lists 1 and 2. In the testing condition, after study of list 1 and the distractor, participants were given 45 sec to recall in any order they wished as many items as they could remember from list 1; next, after study of list 2 and the distractor, participants were given 45 sec to recall in any order they wished as many items as they could remember from list 2. In the restudy condition, participants were instead re-presented the just-studied items of lists 1 and 2 (45 sec each) with new random item presentation order. In both conditions, the distractor after list 3 was followed by an immediate list 3 recall test, in which participants were given 45 sec to recall in any order they wished as many items they could remember from list 3. After the immediate list 3 recall test, lists 2 and 1 were tested in two final recall tests; list 2 was always tested first. Each test took 45 sec. In all recall tests, participants typed in responses on a computer keyboard.

#### Data analysis

On the group level, percent correct recall in the immediate list 3 recall test was examined as a function of experimental condition (testing, restudy) and session (session 1, session 2). In addition, in the testing condition, immediate list recall was examined as a function of list (list 1, list 2, list 3) and session (session 1, session 2). Regarding final recall, percent correct list recall was examined as a function of condition (testing, restudy), list (list 1, list 2), and session (session 1, session 2). Furthermore, number of prior-list intrusions were examined in the immediate list 3 recall test; list 1 and list 2 items that were falsely recalled by participants in the immediate list 3 recall were considered as intrusions. In all analyses of variance (ANOVAs), Greenhouse-Geisser (GG) correction was applied where necessary.

Bayesian statistics were calculated using JASP 0.9.2.0 software (Wagenmakers et al., 2018). *BF*_01_ is reported when the Bayesian analysis provides relatively more evidence for the null hypothesis than for the alternative hypothesis; *BF*_10_ is reported when the analysis provides relatively more evidence for the alternative hypothesis than for null hypothesis.

On the item level, FTE recall and intrusion scores were calculated, separately for sessions 1 and 2. The FTE recall score was calculated by subtracting participants’ immediate list 3 recall rates in the restudy condition from their immediate list 3 recall rates in the testing condition; the FTE intrusion score was calculated by subtracting participants’ number of prior-list intrusions in the restudy condition from their number of prior-list intrusions in the testing condition. Test-retest reliability was estimated with the ICC-2k, which uses a two-way random effects model for absolute agreement (see Hedge et al., 2018; Parsons et al., 2018).

### Results

#### Group level

The results of all recall tests are shown in Table 1. Regarding recall rates in the immediate list 3 recall test, a two-way ANOVA with the within-subjects factors of condition (testing vs. restudy) and session (session 1 vs. session 2) revealed a significant main effect of condition, *F*(1,63) = 36.41, *MSE =* 400.95, *p* < .001, \eta _p^2 = .366, (*BF*_10_ > 100, compared to null model), a significant main effect of session, *F*(1,63) = *7.77, MSE* = 321.90, *p =* .007, \eta _p^2 = .110 (*BF*_10_ = 2.52, compared to null model), and a significant interaction between the two factors, *F*(1,63) = 7.61, *MSE* = 228.17, *p* = .008, \eta _p^2 = .108 (*BF*_10_ = 2.30, compared to two-main-effects model). Thus, while the forward effect on list 3 recall was significant in both sessions, *ps* ≤ .001, the effect was smaller in session 2 than in session 1.

**Table 1:** Results in the Immediate and Final Recall Tests as a Function of Practice Condition and Session in Experiment 1. Means and Standard Errors of the Mean (in Parentheses).

Recall Rates						Intrusions

Session	Test	Condition	List 1	List 2	List 3	List 3

Session 1	Immediate Recall	Testing	66.93 (2.75)	68.88 (2.57)	69.01 (2.56)	0.17 (0.05)
		Restudy			48.70 (3.68)	0.36 (0.10)
		*Difference*			20.31 (3.29)**	*–.19 (.11)*
	Final Recall	Testing	41.28 (4.11)	56.64 (3.54)		
		Restudy	51.69 (3.97)	55.08 (4.00)		
		*Difference*	–10.42 (4.40)*	*1.56 (3.87) *		

Session 2	Immediate Recall	Testing	69.79 (2.93)	68.49 (2.84)	70.05 (2.55)	0.05 (0.03)
		Restudy			60.16 (3.17)	0.19 (0.08)
		*Difference*			9.90 (2.97)**	*–.14 (.08)*
	Final Recall	Testing	38.93 (4.50)	49.61 (4.41)		
		Restudy	58.98 (4.08)	61.98 (3.86)		
		*Difference*	–20.05 (5.53)**	–12.37 (4.89)*		

Pair-wise *t* tests (two sided, uncorrected) for differences between conditions (testing vs. restudy). * p < .05, ** p < .01.

Regarding intrusion rates in the immediate list 3 recall test, a two-way ANOVA with the within-subjects factors of condition (testing vs. restudy) and session (session 1 vs. session 2) showed a significant main effect of condition, *F*(1,63) = 5.56, *MSE* = 0.310, *p* = .022, \eta _p^2 = .081 (*BF*_10_ = 2.18, compared to null model), and a significant main effect of session, *F*(1,63) = 4.26, *MSE* = 0.331, *p* = .043, \eta _p^2 = .063 (*BF*_10_ = 1.29, compared to null model), but no significant interaction between the two factors, *F*(1,63) < 1 (*BF*_01_ = 4.75, compared to two-main-effects model).

Regarding immediate recall of lists 1 to 3 in the testing condition, a two-way ANOVA with the within-subjects factors of list (list 1 vs. list 2 vs. list 3) and session (session 1 vs. session 2) showed no significant main effects or interaction, *Fs* < 1 (*BF*_01_ *s* > 7.00), indicating similar recall rates for lists 1 to 3 in the testing condition in both sessions.

With regard to final recall rates, a three-way ANOVA with the within-subjects factors of condition (testing vs. restudy), list (list 1 vs. list 2), and session (session 1 vs. session 2) was calculated. The analysis showed a significant main effect of condition, *F*(1,63) = 15.35, *MSE* = 887.75, *p* < .001, \eta _p^2 = .196 (*BF*_10_ > 100, compared to null model), a significant main effect of list, *F*(1,63) = 18.23, *MSE* = 461.35, *p* < .001, \eta _p^2 = .224 (*BF*_10_ = 28.83, compared to null model), a significant interaction between the two factors of condition and list, *F*(1,63) = 5.07, *MSE* = 609.66, *p* = .028, \eta _p^2 = .075 (*BF*_10_ = 1.23, compared to three-main-effects model), and a significant interaction between the two factors of condition and session, *F*(1,63) = 4.92, *MSE* = 902.38, *p* = .030, \eta _p^2 = .073 (*BF*_10_ = 3.20, compared to three-main-effects model). Other main effect and interactions were not significant, *Fs* < 1 (*BF*_01_ *s* > 4.00). With respect to the two significant interactions, analyses of single main effects suggested that (i) restudy outperformed testing in the final recall test of list 1, *p* < .001, but not in the final recall test of list 2, *p* = .074, and (ii) restudy outperformed testing in session 2, *p* < .001, but not in session 1, *p* = .142.

#### Individual level

**Figure 2:**
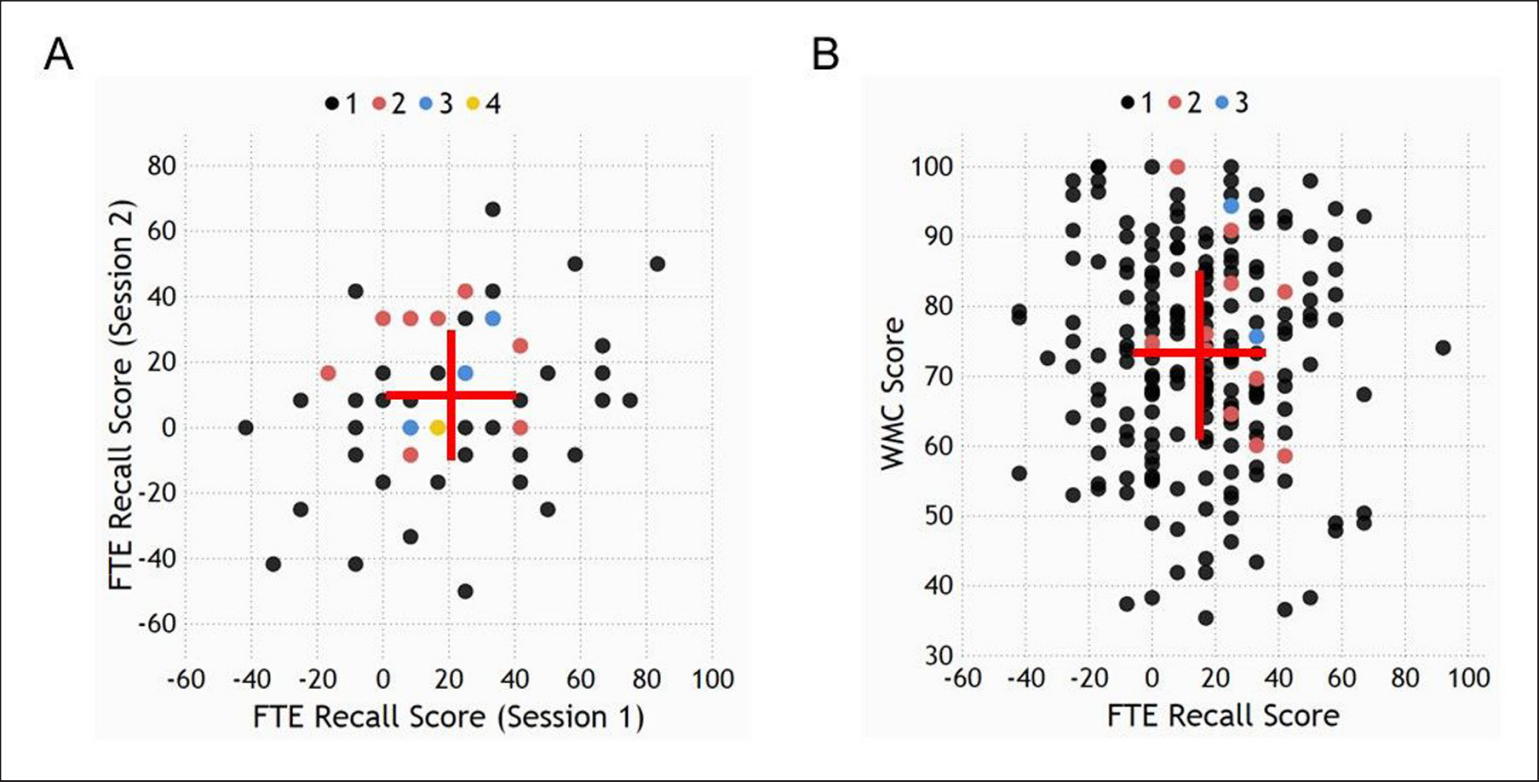
List 3 recall results. **(A)** Scatterplot of the significant test-retest reliability of the FTE recall score, i.e., the difference between participants’ immediate list 3 recall rates in the testing and restudy condition, in Experiment 1. The red marker indicates averaged recall rates from sessions 1 and 2. Error bars show ± 1 standard error of measurement (SEM), which is the square root of the error variance term calculated from the ICC and can be interpreted as the 68% confidence interval for an individual’s data point. Color coding of data points indicates the number of subjects having identical scores on both axes. **(B)** Scatterplot of the nonsignificant correlation between the FTE recall and WMC scores in Experiment 2. Error bars show ± 1 standard error or the mean for the FTE recall and WMC scores, respectively. Color coding of data points indicates the number of subjects having identical scores on both axes.

ICC estimates (and 95% confidence intervals) were calculated separately for FTE recall and intrusion scores between the two sessions. Regarding the FTE recall score, a moderate test-retest reliability was observed, *ICC* = .407, *p* = .013, 95%–CI [.052, .633]. Regarding the FTE intrusion score, no significant test-retest reliability was observed, *ICC* = .016, *p* = .475, 95%–CI [–.634, .406]. A scatterplot of the significant test-retest reliability of the FTE recall score is shown in Figure 2A.

### Discussion

On the group level, the results of Experiment 1 replicate the findings from previous work on the forward testing effect (e.g., Bäuml & Kliegl, 2013; Szpunar et al., 2008; Weinstein et al., 2011; Yang et al., 2019), showing that interim testing of lists 1 and 2, in comparison to restudy of lists 1 and 2, enhances correct recall of list 3 items and reduced the number of prior-list intrusions in the immediate list 3 recall test. Notably, the effect of testing on prior-list intrusions was small and (Bayesian) evidence for the effect was weak. Going beyond the previous research, the results show that the forward effect on list 3 recall rates was reliably reduced in session 2 compared to session 1 (see General Discussion, for a possible explanation of this result).

On the item level, the results of Experiment 1 suggest a moderate test-retest reliability of the FTE recall score, i.e., the difference in correct list 3 recall between the testing and the restudy condition (note, however, that the 95%-CI reached from weak to substantial levels of reliability). In contrast, the reliability of the FTE intrusion score was not significant. Notably, the variance between individuals in the FTE intrusion score was very low (46 participants in session 1 and 54 participants in session 2 had an FTE intrusion score of 0; 37 participants had FTE intrusion scores of 0 in both sessions), which may have decreased the likelihood of observing a significant test-rest reliability. Arguably, a larger test-rest reliability of the FTE intrusion score may be observed with different item materials (see General Discussion).

## Experiment 2

The goal of Experiment 2 was to examine the relationship between the forward testing effect and participants’ WMC. Because the reliability of a measure limits the correlation that can be observed with another measure (Nunally, 1970; Spearman, 1904), based on the results from Experiment 1, only the FTE recall score, but not the FTE intrusion score, was related to participants’ WMC. Experiment 2 took place in a single session. Participants completed the episodic memory task, including both a testing and a restudy condition, first and shortened versions of complex WM span tasks, namely the operation span and symmetry span tasks (Foster et al., 2015), second. As outlined above, no significant correlation between the forward testing effect and participants’ WMC was expected.

### Method

#### Participants

Required sample size was calculated using G*Power (Version 3.1.9.2; Faul, Erdfelder, Buchner, & Lang, 2009). Given *α* = 0.05 and a desired power of 1 – *β* = 0.95 to detect a correlation between FTE recall and WMC scores of medium effect size of |*ρ*| = 0.25 or larger (two-tailed), a required sample size of 197 subjects was calculated. Because data sets from subjects showing an accuracy of less than 80% on the distraction component of one of the working-memory tasks (see below) were discarded from correlational analysis (Conway et al., 2005), a slightly larger sample size was tested.

Two-hundred and forty students from the University of Trier participated in Experiment 2 (mean age: 22.0 years, SD = 3.4 years; 187 females, 53 males). The experiment was carried out in accordance with the recommendations of the Declaration of Helsinki and approved by the local ethical review committee at the University of Trier (reference number: 50/2017). All participants gave written informed consent.

#### Material

The material for the episodic memory task was the same that was used in Experiment 1. For each participant, 72 (out of the 144) items were randomly assigned to six 12-item lists. Three of these lists were used in the testing condition, the other three lists in the restudy condition.

**Figure 3:**
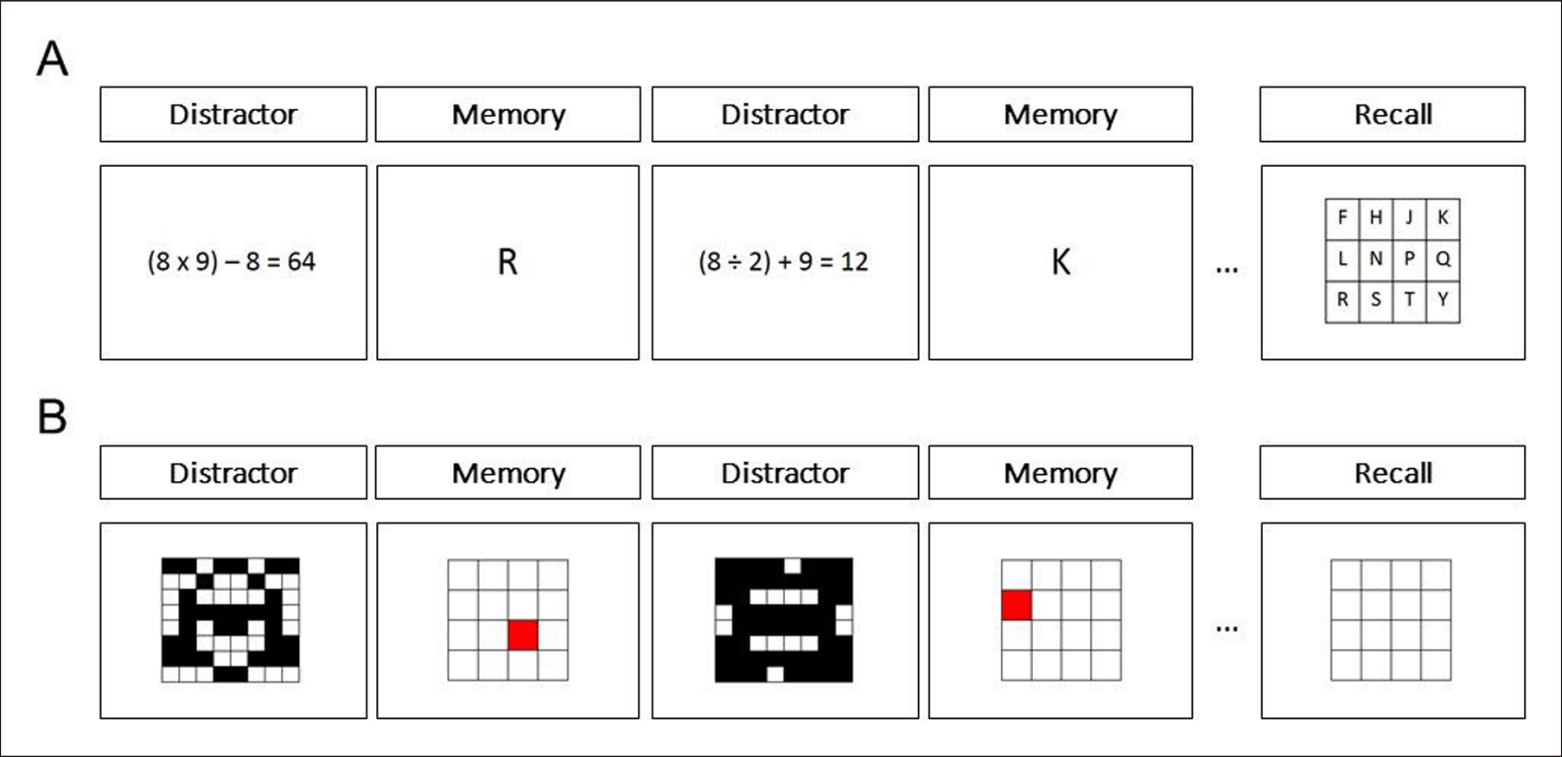
Procedure of the working memory task. **(A)** In the operation span task, trials with to-be-remembered letters and trials with distracting mathematical statements alternated in each sequence. At the end of each sequence, the letters were to be recalled by participants in correct order. Sequence length varied from 3 to 7. **(B)** In the symmetry span task, trials with to-be-remembered locations and trials with distracting symmetry judgments alternated in each sequence. At the end of each sequence, the locations were to be recalled in correct order. Sequence length varied from 2 to 5.

The material for the WMC tasks was taken from Foster et al. (2015), which employed shortened versions of established WM tasks, i.e., the operation span task and the symmetry span task. Both tasks included a memory component and a distraction component. In the operation span task, letters were used as to-be-remembered targets and mathematical statements served as distractors (see Figure 3A). In the symmetry span task, locations of a red square in a 4 × 4 grid were used as to-be-remembered targets and judgments whether or not gridded figures were symmetrical along the vertical axis served as distractors (see Figure 3B).

#### Procedure

The procedure of the episodic memory task was identical to the procedure of the memory task in the first session of Experiment 1, with two exceptions. First, with regard to the 30 sec distractor that followed study of each list, instead of solving simple mathematics, participants counted backward in steps of twos from a random three-digit number. Second, with regard to the 45 sec recall tests, instead of typing in responses on a computer keyboard, participants were asked to write down responses on paper. Different sheets of paper were used for the different recall tests. The distractor and response recording were changed in order to avoid potential overlap with the WMC tasks, especially the operation span task.

After the episodic memory task, two computer-based WMC tasks were conducted employing German translations of the E-Prime 2.0 (SP 2) protocol of Foster et al. (2015). We used model 4 from the study by Foster et al. (2015), including one block of the operation span task and one block of the symmetry span task. Foster et al. (2015) showed that this model predicted participants’ fluid intelligence better than the model using three blocks of the operation span task, indicating high validity of measuring participants’ WMC. Regarding reliability of WMC measures in the study by Foster et al. (2015), Cronbach’s alpha was .690 for the single block of the operation span task and .609 for the single block of the symmetry span task. In the present experiment, the operation span task was conducted first and the symmetry span task second. In the two tasks, trials with to-be-remembered targets, i.e., letters in the operation span task and locations in the symmetry span task, and trials with distractors, i.e., mathematical statements in the operation span task and symmetry judgments in the symmetry span task, appeared alternately in each sequence (see Figure 3). Each sequence ended with a recall test in which the targets were to be recalled in correct order. Sequence length was varied from 3 to 7 in the operation span task and from 2 to 5 in the symmetry span task, and sequences with different lengths were present in random order. Participants completed one block of 5 sequences with different sequence lengths in the operation span task and one block of 4 sequences with different sequence lengths in the symmetry span task, a procedure that has been suggested to have high validity and reliability (see Foster et al., 2015). Sequences with different sequence lengths were randomly sampled in both WMC tasks. Before each block, participants practiced both the memory component and the distraction component of each task. Participants were timed while practicing distraction trials. They were then required to respond within 2.5 SDs of their average response time to each distraction item in each block of the two tasks.

#### Data analysis

Regarding the episodic memory task, percent correct recall in the immediate list 3 recall test was examined as a function of the within-participants factor of experimental condition (testing, restudy). In the testing condition, immediate recall was examined as a function of list (list 1, list 2, list 3). Regarding final recall, percent correct recall was examined as a function of condition (testing, restudy) and list (list 1, list 2). In addition, number of prior-list intrusions was examined in the immediate list 3 recall test. Greenhouse-Geisser (GG) correction was applied where necessary. We note that the results from a serial-position analysis of the immediate list 3 recall data were previously published in a short report (Pastötter, Engel, & Frings, 2018).

Regarding the two working-memory tasks, an absolute partial score was calculated by summing the number of letters (maximum of 25) in the operation span task and the number of locations (maximum of 14) in the symmetry span task that were correctly recalled in correct order for each participant, separately for the two tasks (Turner & Engle, 1989). Next, a relative partial scores was calculated by dividing the absolute partial score by the maximum numbers of recalled targets for each participant, separately for the two tasks. Finally, a combined partial score was calculated by averaging the two relative partial scores for each participant. This combined partial score was considered as participants’ WMC score for correlational analysis. Subjects were discarded from further (WMC and correlational) analyses if accuracy on the distraction component on one of the two tasks (fell below a level of 80% (see Conway et al., 2005).

For the correlational analysis, the FTE recall score was calculated by subtracting participants’ immediate list 3 recall rate in the restudy condition from their list 3 recall rate in the testing condition. The relationship between participants’ FTE recall and WMC scores was analyzed. Shapiro-Wilk testing was used to check the assumption of normal distribution for the two scores. The assumption of normality had to be rejected for both scores, *W*_240_ *s* ≤ .985, *ps* ≤ 0.026. Therefore, Kendall’s tau correlations (*r_τ_*) were calculated to examine the relationship between FTE recall and WMC scores.

Bayesian statistics were calculated using JASP 0.9.2.0 software (see Wagenmakers et al., 2018). *BF*_01_ is reported when the Bayesian analysis provides relatively more evidence for the null hypothesis than for the alternative hypothesis; *BF*_10_ is reported when the analysis provides relatively more evidence for the alternative hypothesis than for null hypothesis.

### Results

#### Episodic memory task

The results of the episodic memory task are shown in Table 2. In the immediate list 3 recall test, participants correctly recalled significantly more list 3 items in the testing condition than in the restudy condition,*t*_239_ = 10.55, *p* < .001, *d* = .681 (*BF*_10_ > 100). In addition, participants produced fewer prior-list intrusions in the testing condition than in the restudy condition, *t*_239_ = 4.51, *p* < .001, *d* = .291 (*BF*_10_ > 100). Together, these results suggest a significant forward testing effect.

**Table 2:** Results in the Immediate and Final Recall Tests as a Function of Practice Condition in Experiment 2. Means and Standard Errors of the Mean (in Parentheses).

Recall Rates					Intrusions

Test	Condition	List 1	List 2	List 3	List 3

Immediate Recall	Testing	66.32 (1.24)	63.58 (1.35)	64.72 (1.44)	0.10 (0.02)
	Restudy			49.13 (1.85)	0.38 (0.06)
	*Difference*			15.59 (1.48)**	–.29 (.06)**
Final Recall	Testing	37.57 (1.86)	45.80 (1.80)		
	Restudy	52.26 (1.88)	55.52 (1.90)		
	*Difference*	–14.69 (2.05)**	–9.72 (1.91)**		

Pair-wise *t* tests (two sided) for differences between conditions (testing vs. restudy). ** *p < .01*

Regarding immediate recall of lists 1 to 3 in the testing condition, a one-way ANOVA with the within-subjects factor of list (list 1 vs. list 2 vs. list 3) showed no significant effect, *F*(2,478) = 2.38, *MSE* = 194.61, *p* = .095, G-G corrected (*BF*_01_ = 6.52, compared to null model), indicating that recall was equally successful from lists 1 to 3 in the testing condition.

Regarding final recall testing, a two-way ANOVA with the within-subjects factors of condition (testing vs. restudy) and list (list 1 vs. list 2) showed a significant main effect of condition, *F*(1,239) = 72.72, *MSE* = 491.62, *p* < .001, \eta _p^2 = .233 (*BF*_10_ > 100, compared to null model) a significant main effect of list, *F*(1,239) = 17.63, *MSE* = 449.68, *p* < .001, \eta _p^2 = .069 (*BF*_10_ > 100, compared to null model), but no significant interaction between the two factors, *F*(1,239) = 3.26, *MSE* = 453.70, *p* = .072, \eta _p^2 = .013 (*BF*_01_ = 2.191, compared to two-main-effects model). Thus, both lists were recalled better in the restudy than in the testing condition.

#### Working memory tasks

The data sets from 23 subjects were discarded from WMC and correlational analysis due to accuracy lower than 80% on the distraction component of either the operation span task (15 subjects) or the symmetry span task (8 subjects). The data from one more subject was discarded due to system error during accomplishment of the operation span task. Thus, the data of 216 subjects went into further analysis.

Subjects’ mean absolute partial score was 18.53 (SD = 4.83; range 2–25) in the operation span task and 10.14 (SD = 2.70; range 4–14) in the symmetry span task. Mean relative partial scores were 0.74 (SD = 0.19; range 0.08–1.00) in the operation span task and 0.72 (SD = 0.19; range 0.29–1.00) in the symmetry span task. Partial scores in the two tasks were correlated, *r_τ_* = .183, *p* < .001 (*BF*_10_ > 100). The mean combined partial score (WMC score) was 0.73 (SD = 0.15, range 0.35–1.00, median: 0.74).

#### Relationship between FTE recall and WMC scores

The relationship between FTE recall and WMC scores is depicted in a scatterplot in Figure 2B. The correlation between the two scores was not significant, *r_τ_* = -.018, *p* = .715 (*BF*_01_ = 10.43). Additional median split analysis (based on participants’ WMC score) confirmed that the forward testing effect in list 3 recall was equally present in low-WMC (mean: 15.43%, SD = 22.09%) and high-WMC participants (mean: 16.28%, SD = 24.42%), *t*_214_ < 1 (*BF*_01_ = 6.51). In contrast to the FTE recall score, participants’ mean immediate list 3 recall rates (averaged over testing and restudy conditions) was significantly related to participants’ WMC score, *r_τ_* = .104, *p* = .026.

### Discussion

With regard to the episodic memory task, the results of Experiment 2 replicate the findings from Experiment 1 showing enhanced list 3 recall and fewer prior-list intrusions in the immediate list 3 recall test in the testing compared to the restudy condition. More importantly, with regard to the analysis of individual differences in the forward testing effect, the results showed no correlation between FTE recall and WMC scores, suggesting that the forward testing effect does not depend on participants’ WMC.

## General Discussion

On the group level, the results of both experiments demonstrate a significant forward testing effect, with interim testing of lists 1 and 2 enhancing recall of list 3 and reducing the number of prior-list intrusions in the immediate list 3 recall test. On the individual level, the results of Experiment 1 show that the forward effect on list 3 recall has moderate test-retest reliability between two experimental sessions. In addition, the results of Experiment 2 demonstrate that the forward effect on list 3 recall does not depend on participants’ WMC. These results suggest that the forward testing effect is a reliable phenomenon that does not depend on learner characteristics concerning WMC.

The present study adds to earlier research that also took an individual-differences approach in studying the benefits of retrieval-based learning. Developmental and clinical research has established both direct and indirect testing effects in children (Aslan & Bäuml, 2016; Karpicke et al., 2016; Lipowski et al., 2014), healthy older adults (Coane, 2013; Pastötter & Bäuml, 2019; Meyer & Logan, 2013), and patients with memory impairment (Balota et al., 2006; Pastötter et al., 2013; Sumowski, Wood et al., 2010). In addition, several studies examined the influence of participants’ WMC on the backward testing effect (Agarwal et al., 2017; Aslan & Bäuml, 2011) and test-potentiated learning (e.g., Brewer & Unsworth, 2012; Wiklund-Hörnqvist et al., 2014). These studies found no (direct) relationship between WMC and retrieval-based learning. The present study adds to this research by showing that the forward testing effect is also not (directly) related to participants’ WMC. Whether there are indirect relationships between the forward testing effect and participants’ WMC, i.e., whether the relationship can be moderated by other variables, needs to be tested in future work. Indeed, such moderation has been reported for test-potentiated learning, with test anxiety as moderator (Tse & Pu, 2012). In addition, future research may like to examine the association of WMC to list isolation effects arising from different forms of memory retrieval, e.g., episodic, semantic, and short-term memory retrieval (for related research, see Wahlheim, Alexander, & Kane, 2019), in comparison to non-retrieval baseline conditions.

In the literature, both encoding and retrieval mechanisms have been suggested to contribute to the forward testing effect. Specifically, regarding encoding, testing between the study of lists has been suggested to induce encoding resets (Pastötter et al., 2018; Pastötter et al., 2011) and/or a change in participants’ encoding strategy (Chan et al., 2018; Wissman et al., 2011). In addition, regarding retrieval, testing has been argued to promote contextual list segregation, enhancing list differentiation and reducing interference between lists at test (Bäuml & Kliegl, 2013; Szpunar et al., 2008; see also Wahlheim, 2015, for an integration account). The present study was not designed to test theoretical accounts against each other. In fact, the finding that, in both experiments, the three lists showed the same immediate recall rates in the testing condition is consistent with both reset-of-encoding and interference-reduction views on the forward effect. In addition, the finding that, in Experiment 1, the forward effect on list 3 recall was found to be reduced in session 2 compared to session 1 supports the strategy-change view on the effect. Indeed, participants may have changed encoding strategy after testing in session 1 and thus used a more elaborative encoding strategy in both the testing and the restudy condition in session 2, which may have the forward testing effect in session 2. Thus, on the group level, the present results are consistent with the idea that multiple mechanisms may contribute to the forward effect. On the individual level, the present findings suggest that the forward testing effect is unrelated to participants’ WMC, indicating that the underlying (encoding or retrieval) mechanism(s) may not depend on WM characteristics.

In recent work, it was speculated that the forward testing effect may partly be mediated by some form of self-induced forget instruction, inducing participants to think that the practiced information is no longer needed and thus can be forgotten (Abel & Bäuml, 2016; Szpunar, McDermott, & Roediger, 2007). In fact, intentional forgetting of previously studied, old information (e.g., list 1) can lead to enhancement of subsequently studied, new information (e.g., list 2), as has been demonstrated in the list-method directed forgetting (LMDF) task (for a review, see Pastötter et al., 2017). In this task, both encoding and retrieval factors have been suggested to contribute to the enhancement effect, with the relative contribution of the factors depending on list recall order at final test (Pastötter et al., 2012). Regarding individual differences, the list 2 enhancement effect has been shown to be unrelated to WMC when list 2 is recalled after list 1 (Delaney & Sahakyan, 2007) and mainly encoding factors (i.e., reset of encoding) contribute to the effect (Pastötter et al., 2012), but is positively related to WMC when list 2 is recalled first (Aslan, Zellner, & Bäuml, 2010) and mainly retrieval factors (i.e., interference reduction) contribute to the effect (Pastötter et al., 2012). In the present study, the target list (list 3) was always recalled first after encoding. Still, the forward testing effect was unrelated to participants’ WMC. These findings suggest that the forward testing effect may differ mechanistically from the enhancement effect in LMDF. Indeed, encoding factors (e.g., reset of encoding, change of encoding strategy, more attentional and motivational encoding) may play a much greater role for the forward testing effect than they do for the enhancement effect in LMDF.

In Experiment 1, the FTE intrusion score, i.e., the forward effect on incorrect prior-list intrusions in the immediate list 3 recall test, was not found to be reliable. Because the reliability of a measure limits the correlation that can be observed with another measure (Nunally, 1970; Spearman, 1904), the relationship between the FTE intrusion score and the WMC score thus was not examined in Experiment 2. Arguably, the variance between individuals in the FTE intrusion score was very low in the present study. Therefore, larger test-retest reliability of the FTE intrusion score might be observed with larger variance between individuals. Future studies may like to examine this issue when using item materials that produce more intrusions and thus larger variance between subjects. For example, Szpunar et al. (2008) let participants study semantically related word lists that were composed of different words from the same categories. With such interrelated item material, variance between individuals in the FTE intrusion score may be increased and larger test-retest reliability may be observed.

Regarding final recall of lists 1 and 2, the results of both experiments revealed benefits of restudy over testing, which were larger for list 1 then for list 2 (in Experiment 1). Critically, final list 1 recall provided only a rough measure of the backward testing effect (with retroactive interference from lists 2 and 3 and, when prior recall of lists 3 and 2), whereas final list 2 recall provided a mixed measure of backward and forward effects (with retroactive interference from lists 3 and prior recall of list 3). Therefore, individual differences in final recall of lists 1 and 2 between the testing and restudy condition were not further analyzed with regard to test-rest reliability or relationship to participants’ WMC. Notably, regarding the backward testing effect in final list 1 recall, the finding that restudied items were better recalled than previously tested items is consistent with the literature, showing that the backward testing effect is most prominent when the final recall testing is administered after a relatively long delay of several days or weeks, but is often eliminated or even reversed when final recall testing is administered after a relatively short delay of several minutes (see Kornell, Bjork, & Garcia, 2011; Roediger & Karpicke, 2006b).

To conclude, the results of two experiments suggest that the FTE is a reliable phenomenon that is not related to individuals’ WMC. Together with the findings from previous work on the backward testing effect and test-potentiated learning, the present results suggest that the effectiveness of retrieval-based learning does not depend to a significant degree on learners’ WMC. Future work may examine the relationship of the benefits of retrieval-based learning to other learner characteristics, e.g., general fluid intelligence. Following the view that WMC is an explanatory construct for persons’ intellectual abilities that predicts reasoning abilities and general fluid intelligence to a very large degree (Oberauer, Schulze, & Wilhelm, 2005), we would expect that the benefits of testing are broadly present in learners’ with different cognitive abilities.

## Data Accessibility Statement

Item material and data can be found at Open Science Framework, DOI: https://doi.org/10.17605/OSF.IO/TFP4S.
